# Skeletal Softening in *Cyprinus carpio rubrofuscus*: Insights from Mineral Metabolism, Histology, and Autophagy

**DOI:** 10.3390/ani16101448

**Published:** 2026-05-09

**Authors:** Wan Fan, Zaixuan Zhong, Qingheng Wang, Jiajia Fan, Yuanyuan Tian, Zicheng Zhu, Huaping Zhu, Dongmei Ma

**Affiliations:** 1 Fisheries College, Guangdong Ocean University, Zhanjiang 524088, China; 2 Pearl River Fisheries Research Institute, Chinese Academy of Fishery Sciences, Guangzhou 510380, China; 3 Key Laboratory of Tropical and Subtropical Fishery Resources Application and Cultivation, Ministry of Agriculture and Rural Affairs, Guangzhou 510380, China; 4 Key Laboratory of Aquatic Animal Immunology and Sustainable Aquaculture of Guangdong Province, Guangzhou 510380, China

**Keywords:** softened-bone *C. c. rubrofuscus*, bone deformation, mineral metabolism, osteocytes, autophagy

## Abstract

*C. c. rubrofuscus* is an economically important species in South China. During breeding, some individuals develop skeletal softening, but the underlying mechanisms remain unclear. In this study, five-month-old *C. c. rubrofuscus* were classified into a hard-bone group and a softened-bone group based on X-ray radiography. A systematic comparison was then conducted between the two groups, including analyses of external morphology, skeletal morphology, vertebral bone mineral density (BMD), calcium (Ca) and phosphorus (P) levels in serum and bone tissue, bone histology, and the expression of autophagy-related genes and proteins. The prevalence of the softened-bone phenotype was approximately 14% in the cultured population. Compared with the hard-bone group, the softened-bone group showed significantly lower vertebral BMD, significantly increased serum Ca levels, significantly decreased serum P levels, and markedly reduced Ca and P contents in bone tissue. In addition, varying degrees of deformity were observed in the ribs, caudal intermuscular spines, and urostyle. Histological examination further revealed severe skeletal malformations in the softened-bone group, characterized by irregular cortical bone thickness in the ribs and pterygiophores, together with a significantly reduced osteocyte density. Meanwhile, microtubule-associated protein 1 light chain 3 (LC3) levels were significantly increased, whereas sequestosome 1 (p62) levels were significantly decreased. The upregulated expression of autophagy-related genes suggested dysregulated autophagy, which may contribute to osteocyte loss. Collectively, these results indicate that the softened-bone phenotype in *C. c. rubrofuscus* is not associated with obvious changes in external body morphology. However, disrupted Ca-P homeostasis, together with altered autophagy, may impair osteocyte viability and bone mineralization, ultimately leading to bone softening and skeletal deformity. These findings provide a theoretical basis for further investigation of the mechanisms underlying bone softening and deformity in *C. c. rubrofuscus* and for the genetic improvement and selective breeding of cyprinid fish to reduce the occurrence of the softened-bone trait.

## Simple Summary

Bone softening and deformity are closely associated with disorders of mineral metabolism. As essential elements for bone mineralization, calcium and phosphorus directly influence skeletal development. Elucidating the biological and genetic basis of bone softening in *Cyprinus carpio rubrofuscus* (*C. c. rubrofuscus*) is important for breeding. In this study, hard-bone and softened-bone fish were compared in terms of morphology, bone histology, bone mineral density (BMD), serum and bone calcium and phosphorus levels, and the expression of autophagy-related genes and proteins. The softened-bone group showed significantly lower vertebral BMD, higher serum calcium, lower serum phosphorus, and reduced calcium and phosphorus levels in bone. Histological analysis revealed severe deformities of the ribs and pterygiophores, accompanied by reduced osteocyte density. In addition, increased levels of microtubule-associated protein 1 light chain 3 (LC3) and decreased levels of protein sequestosome 1 (p62), together with the upregulated expression of autophagy-related genes (*ulk1*, *pik3c3*, *atg5*, *atg7*, *atg12*, *lc3b*, and *p62*), indicated dysregulated autophagy, which may contribute to osteocyte loss. These findings suggest that disrupted calcium-phosphorus metabolism and autophagy may jointly induce bone softening by impairing osteocyte viability and mineralization, thereby providing a theoretical basis for understanding its underlying mechanisms and supporting genetic improvement and selective breeding in cyprinid fish.

## 1. Introduction

*Cyprinus carpio rubrofuscus* (*C. c. rubrofuscus*) is a traditional aquaculture species widely cultured in rice-fish farming systems in South China. Owing to its tender flesh, desirable flavor, high nutritional value, and strong disease resistance, *C. c. rubrofuscus* has become a high-quality species for rice-field aquaculture [1,2]. However, our previous investigations found that some individuals developed curved and softened bones during artificial breeding. These individuals are referred to as softened-bone *C. c. rubrofuscus*. Notably, after steaming or boiling, the bones of softened-bone fish become tender and edible, and the intermuscular spines are barely perceptible, which has increased market preference for this phenotype. However, the mechanisms underlying bone softening and deformity in *C. c. rubrofuscus* remain largely unclear.

Bones play essential roles in maintaining body morphology, supporting locomotion, and protecting internal organs in fish. During development, the skeleton is susceptible to deformities caused by environmental, nutritional, and genetic factors [3]. Previous studies have shown that skeletal deformities can directly impair fish physiology, leading to reduced swimming ability, slower growth, and decreased vitality [4,5]. Bone development and skeletal deformities have been extensively studied in several economically important fish species, including *Cynoglossus semilaevis*, *Hippoglossus hippoglossus*, *Trachinotus ovatus*, and *Lutjanus* spp. [6,7,8,9]. For example, in juvenile *T. ovatus*, the incidence of skeletal deformities increases as water temperature rises above 26 °C [10]. In Nong’an County, Jilin Province, approximately 70% of carp raised in newly established ponds on saline-alkali land develop osteomalacia characterized by spinal curvature and rib deformities. Researchers have suggested that the depletion of available phosphorus in aquaculture water after flowing through saline-alkali land contributes to these bone abnormalities [11]. In addition, reduced calcium and phosphorus levels in feed can induce vertebral fusion and caudal skeletal deformities in *C. c. rubrofuscus* [12]. Collectively, these findings indicate that both water conditions and dietary mineral composition can affect skeletal development.

In addition to external factors, genetic factors are also essential for normal skeletal development. For example, CATSHL syndrome follows either autosomal dominant or autosomal recessive inheritance. In Danio rerio, knockout of the *fgfr3* gene leads to craniofacial skeletal deformities [13]. In Atlantic salmon, the incidence of skeletal deformities in triploid offspring ranges from 30% to 35%, compared with 8% to 30% in diploid offspring [14]. Despite these advances, studies on skeletal deformities in carp remain limited. It is still unclear whether bone softening and deformity in *C. c. rubrofuscus* affect individual growth and what mechanisms underlie these abnormalities. We hypothesized that the abnormal skeletal phenotype observed in softened-bone *C. c. rubrofuscus* is not merely an external morphological variation, but rather a manifestation of skeletal softening and deformity caused by disrupted bone metabolism. In the present study, we compared body size, skeletal morphology, calcium and phosphorus levels, and bone histological structure between hard-bone and softened-bone *C. c. rubrofuscus*. Our aim was to investigate the causes of bone bending and deformity in the softened-bone phenotype. The findings are expected to provide a theoretical foundation for the selective breeding of new softened-bone *C. c. rubrofuscus* varieties.

## 2. Materials and Methods

### 2.1. Experimental Materials

All experimental fish were obtained from the Fangcun Aquaculture Base of the Pearl River Fisheries Research Institute, Chinese Academy of Fishery Sciences. The fry were initially reared in net cages and subsequently transferred to the institute’s aquaculture base for grow-out. During the first two weeks after hatching, the fry were fed newly hatched fairy shrimp. Thereafter, a formulated compound diet was provided daily from 9:00 a.m. to 6:00 p.m. to apparent satiation. The feed ingredients and proximate composition are shown in Table 1.

A total of 120 *C. c. rubrofuscus* were randomly sampled at 5 months of age. All fish were examined by X-ray imaging, and BMD was measured using an in vivo X-ray imaging system (KUBTEC Scientific, Stratford, CT, USA). Individuals with normal rib morphology and a mean vertebral BMD > 0.02 g/cm^2^, calculated from the average values of the 1st, 5th, and 10th vertebrae from the caudal end, were classified into the hard-bone group. Individuals with markedly curved ribs and a mean vertebral BMD < 0.02 g/cm^2^ were classified into the softened-bone group. Based on these criteria, 17 fish (14%) were assigned to the softened-bone group, and the remaining 103 fish (86%) were assigned to the hard-bone group.

### 2.2. Experimental Methods

#### 2.2.1. Morphological Trait Analysis

Twelve fish were randomly selected from each of the hard-bone and softened-bone groups for subsequent experiments. For each fish, nine growth-related morphological traits were measured: total length (T_L_), standard length (S_L_), body depth (B_D_), body width (B_W_), head length (H_L_), head height (H_H_), caudal peduncle depth (CP_D_), caudal peduncle length (CP_L_), and caudal fin length (CF_L_). To minimize the effect of body size, the following ratios were calculated: T_L_/S_L_, B_W_/S_L_, B_D_/S_L_, H_L_/S_L_, H_H_/B_D_, CP_L_/S_L_, CP_D_/CP_L_, and CF_L_/S_L_.

#### 2.2.2. Bone Densitometry

An in vivo X-ray imaging system was used to acquire radiographs and measure BMD in experimental fish. The system is based on differential attenuation of high- and low-energy X-ray beams as they pass through bone tissue. Twelve experimental fish were randomly selected from each group (*n* = 12). BMD was measured at the 1st, 5th, and 10th vertebrae in both the hard-bone and softened-bone groups.

#### 2.2.3. Measurement of Calcium and Phosphorus Levels in Blood and Bones

Twelve fish were randomly selected from each group, and blood samples were collected from the caudal artery (*n* = 12). After standing, the samples were centrifuged at 2000× *g* for 15 min at 4 °C using a refrigerated centrifuge. The serum was then separated and stored at −20 °C for subsequent calcium and phosphorus assays. Opercular bone samples were placed in cryogenic tubes and stored at −80 °C. After grinding in liquid nitrogen, the bone homogenates were used for calcium and phosphorus determination. The following commercial kits were used: Blood Calcium Concentration Detection Kit (Solarbio, Beijing, China), Blood Phosphorus Concentration Detection Kit (Solarbio, Beijing, China), Tissue Total Phosphorus Level Detection Kit (Solarbio, Beijing, China), and Calcium Detection Kit (Solarbio, Beijing, China). All assays were performed strictly in accordance with the manufacturers’ instructions.

#### 2.2.4. Bone Staining

Alizarin Red S staining was performed to visualize skeletal structures. Two fish were randomly selected from each group and fixed overnight in 4% paraformaldehyde. After fixation, the samples were briefly immersed in 1% potassium hydroxide (KOH), and the scales and skin were carefully removed. The specimens were then stained in Alizarin Red S solution (saturated Alizarin Red S in ethanol: 0.5% KOH = 1:9, *v*/*v*) for 48 h. After staining, the solution was discarded, and excess dye was rinsed off with distilled water. The samples were subsequently bleached in a freshly prepared solution of 3% hydrogen peroxide and 2% KOH (1:1, *v*/*v*) at room temperature for 20 min. Following bleaching, the specimens were cleared through a graded glycerol series prepared with 0.5% KOH (20%, 40%, 60%, and 80% glycerol) until the body became transparent. The cleared specimens were finally transferred to 100% glycerol for long-term preservation and then photographed for morphological observation.

#### 2.2.5. Histomorphological Analysis of Bone

Section preparation:

Three fish were randomly selected from each group (hard-bone and softened-bone groups). Bone-containing tissues were collected from the upper rib region adjacent to the vertebral column and from the distal region of the dorsal fin rays, with a thin layer of attached muscle retained. The samples were fixed overnight in 4% paraformaldehyde and decalcified in EDTA solution until the tissue could be penetrated by a needle without resistance. After decalcification, the samples were dehydrated through a graded ethanol series, cleared in xylene, and embedded in paraffin. Cross-sections were cut at 7 μm using a microtome, dried, and stored at room temperature.

Hematoxylin and eosin (H&E) staining:

For each experimental fish, three consecutive sections were selected, and the mean value from these sections was used as one biological replicate for statistical analysis (*n* = 3). Paraffin sections were deparaffinized, rehydrated, stained with hematoxylin, differentiated, and rinsed under running water. The sections were then counterstained with eosin, dehydrated through a graded ethanol series (including two changes in absolute ethanol and a final dehydration in fresh absolute ethanol), cleared in xylene, and mounted with neutral resin. Whole-slide imaging was performed using a digital slide scanner (NanoZoomer^®^ S360, Hamamatsu Photonics, Deutschland, Germany), and the images were examined and analyzed using NDP.view 2.9.22 RUO (Hamamatsu Photonics, Deutschland, Germany). To systematically evaluate skeletal morphological variations, we quantitatively analyzed cortical bone on histological sections. The maximum and minimum cortical bone widths in the target skeletal element were measured on each section, and their difference was calculated as an indicator of cortical bone thickness heterogeneity. Consecutive sections from each fish were measured, and the mean value across sections served as the final representative value for that individual. A larger difference indicates greater spatial heterogeneity in cortical bone thickness, reflecting more severe abnormalities in bone mineralization or growth patterns; thus, this value was used as a quantitative index of skeletal deformity severity. For osteocyte density analysis, the region of interest was delineated within the cortical bone area on selected sections, and the total cortical bone area was precisely measured. All visible osteocytes (i.e., cells within lacunae) in this region were counted, and osteocyte density was calculated as the number of osteocytes per unit cortical bone area. The mean value from multiple sections of each fish was used for subsequent statistical analysis. This parameter reflects bone metabolic activity or the dynamic balance between bone formation and resorption.

#### 2.2.6. Autophagy-Related Protein Measurement by ELISA

Serum levels of p62 and LC3 were measured using the same serum samples described in Section 2.2.3 (*n* = 12). The following ELISA kits were used: Danio rerio Microtubule-Associated Protein LC3 ELISA Research Kit (Welab, Beijing, China) and Danio rerio p62 Protein ELISA Research Kit (Welab, Beijing, China). All assays were performed strictly in accordance with the manufacturer’s instructions. The resulting ELISA data were used as exploratory evidence to support the overall trend of autophagy-related protein changes, rather than as rigorously validated absolute quantification in *C. c. rubrofuscus*.

#### 2.2.7. qPCR Analysis of Autophagy-Related Gene Expression in Bone Tissue

Fish were fasted for 24 h and anesthetized with 100 mg/L MS-222 (Sigma, St. Louis, MO, USA). Nine individuals were randomly selected from each group, and opercular bone tissues were rapidly excised. The surface skin and attached tissues were carefully removed, and the samples were immediately frozen in liquid nitrogen and stored at −80 °C until further use. For each group, three samples were pooled and treated as one biological replicate for RNA extraction, resulting in a total of three biological replicates (*n* = 3). Total RNA was extracted using the EASYspin Plus Bone Tissue RNA Rapid Extraction Kit (Aidlab, Beijing, China) according to the manufacturer’s instructions. RNA purity and concentration were determined using a NanoDrop 2000 spectrophotometer (Thermo Fisher Scientific, Waltham, MA, USA), and RNA integrity was assessed using an Agilent 2100 Bioanalyzer (Agilent Technologies, Santa Clara, CA, USA). Total RNA from each replicate was reverse-transcribed into complementary DNA (cDNA) using the PrimeScript RT Reagent Kit with gDNA Eraser (Takara, Dalian, China). Gene-specific primers for the autophagy-related genes (*ulk1*, *pik3c3*, *atg5*, *atg7*, *atg12*, *lc3b*, and *p62*) and the reference gene glyceraldehyde-3-phosphate dehydrogenase (GAPDH) were designed using Primer Premier v5.0, and the primer sequences are listed in Table 2. Quantitative real-time PCR (qPCR) was performed on a 7500 Real-Time PCR System (Applied Biosystems, Carlsbad, CA, USA) in a total reaction volume of 20 μL, containing 10 μL of 2 × SYBR Green qPCR Premix, 0.4 μL of forward primer, 0.4 μL of reverse primer, 2 μL of cDNA template, and 7.2 μL of nuclease-free water. The amplification conditions were as follows: initial denaturation at 95 °C for 30 s, followed by 35 cycles of 95 °C for 10 s, 60 °C for 10 s, and 72 °C for 30 s. Each sample was analyzed with three technical replicates and three biological replicates. The relative expression levels of the target genes were calculated using the 2^−ΔΔCt^ method, with GAPDH as the internal reference gene.

#### 2.2.8. Statistical Analysis

All data are expressed as the mean ± standard deviation (SD). Statistical analyses were conducted using R version 4.5.0. Data distribution was first assessed for normality using the Shapiro–Wilk test, and homogeneity of variance was evaluated using Levene’s test. For comparisons between the hard-bone and softened-bone groups, an independent-samples *t*-test was used when the assumptions of normality and equal variance were satisfied; otherwise, Welch’s *t*-test was applied. Differences were considered statistically significant at *p* < 0.05.

## 3. Results

### 3.1. Comparative Analysis of Body Morphology

Based on the eight morphometric ratio indices presented in Table 3 and Table 4, no significant differences in body morphology were detected between the softened-bone and hard-bone groups of *C. c. rubrofuscus* at 5 months of age (*p* > 0.05).

### 3.2. Comparative Analysis of Bone Mineral Density

**Figure 1:**
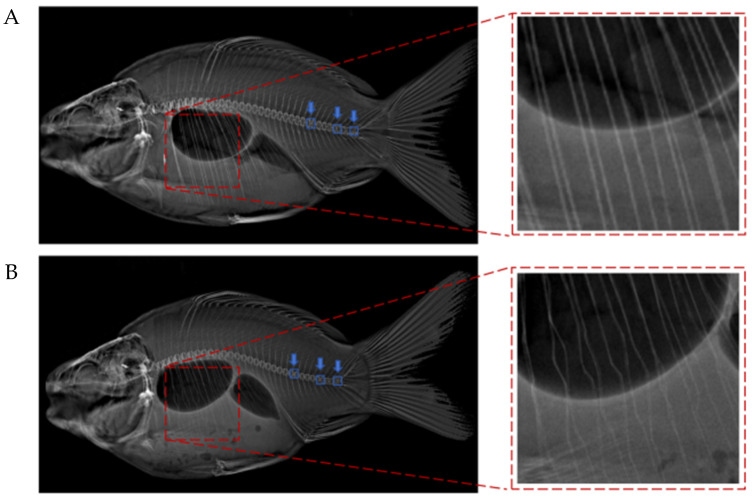
X-ray images two *C. c. rubrofuscus* groups. (**A**) X-ray image of a representative hard-bone *C. c. rubrofuscus* specimen, with an enlarged view of the ribs; blue arrows indicate the BMD measurement sites at the 1st, 5th, and 10th vertebrae. (**B**) X-ray image of a representative softened-bone *C. c. rubrofuscus* specimen, with an enlarged view of the ribs.

**Figure 2:**
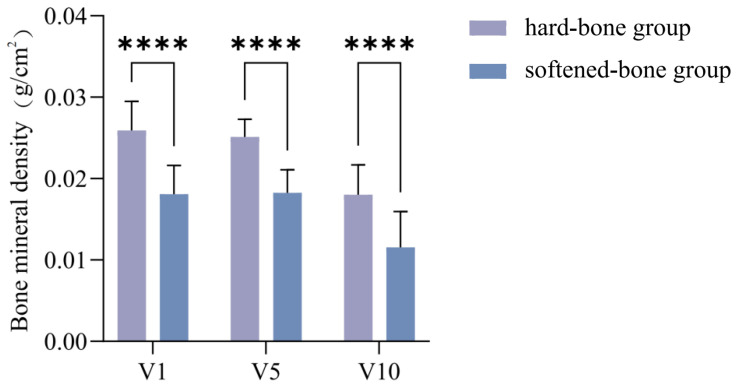
Comparison of b BMD between two groups of *C. c. rubrofuscus*. Vertebral BMD was measured at three sites (the 1st, 5th, and 10th vertebrae from the caudal end). Data are presented as mean ± SD (*n* = 12). **** indicates *p* < 0.0001.

X-ray images of the hard-bone and softened-bone groups (Figure 1A,B) showed marked rib curvature in the softened-bone *C. c. rubrofuscus*. Dual-energy X-ray absorptiometry (DXA) indicated that the BMD values of the 1st, 5th, and 10th vertebrae in the hard-bone group were 0.026 ± 0.004, 0.025 ± 0.002, and 0.018 ± 0.004 g/cm^2^, respectively. The corresponding values in the softened-bone group were 0.018 ± 0.004, 0.018 ± 0.003, and 0.012 ± 0.004 g/cm^2^, respectively. As shown in Figure 2, BMD was significantly higher in the hard-bone group than in the softened-bone group across all examined vertebral segments (*p* < 0.0001).

### 3.3. Comparative Analysis of Alizarin Red Staining

**Figure 3:**
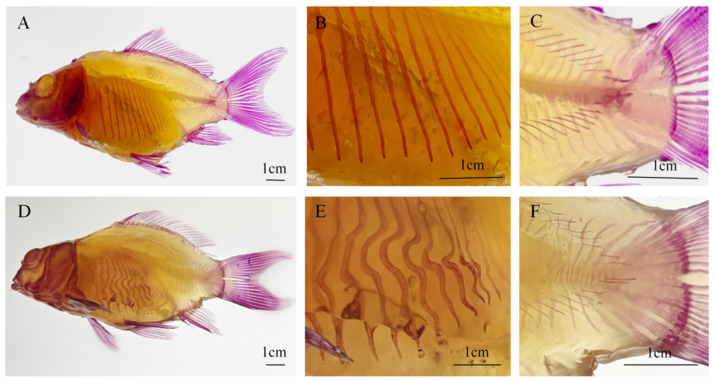
Comparison of Alizarin red-stained between the two groups. (**A**) Alizarin Red S staining of a representative hard-bone *C. c. rubrofuscus* specimen. (**B**) Ribs of a representative hard-bone specimen. (**C**) Intermuscular spines and urostyle of a representative hard-bone specimen. (**D**) Alizarin Red S staining of a representative softened-bone *C. c. rubrofuscus* specimen. (**E**) Ribs of a representative softened-bone specimen. (**F**) Intermuscular spines and urostyle of a representative softened-bone specimen.

Alizarin Red S staining (Figure 3) revealed clear skeletal differences between the two groups. In the hard-bone group, the ribs were relatively straight, uniformly thick, and smooth in surface morphology (Figure 3B). In contrast, softened-bone *C. c. rubrofuscus* exhibited markedly curved ribs with deformed, thickened, and flattened bony structures (Figure 3E). In the hard-bone group, the intermuscular spines showed a uniform morphology, and the urostyle exhibited consistent thickness and a regular shape (Figure 3C). By contrast, in the softened-bone group, the intermuscular spines showed varying degrees of curvature, whereas the urostyle exhibited obvious deformity and uneven thickness (Figure 3F). In addition, the Alizarin Red S staining intensity was lower in the softened-bone group than in the hard-bone group, suggesting reduced mineralization (Figure 3).

### 3.4. Comparative Analysis of Calcium and Phosphorus Levels in Serum and Bone Tissues

**Figure 4:**
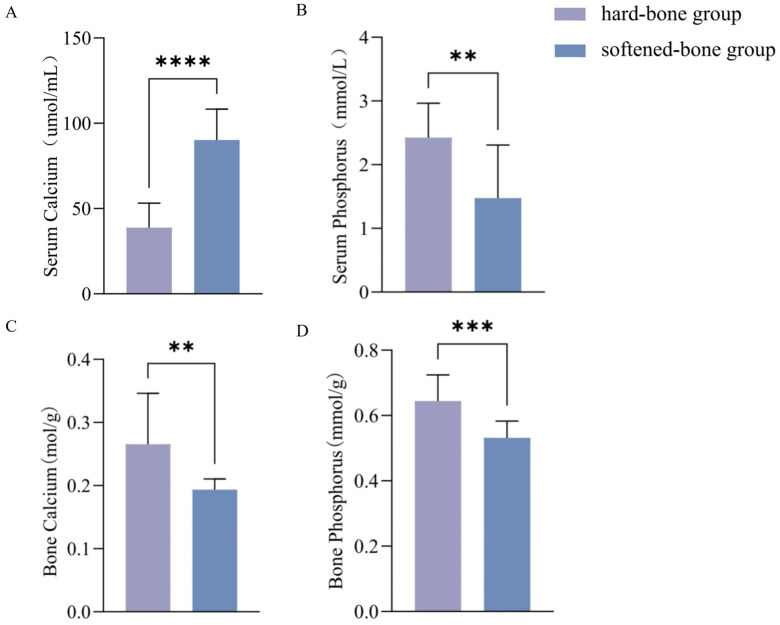
Comparison of calcium and phosphorus levels in serum and bone tissues between the two groups. (**A**) Serum calcium; (**B**) Serum phosphorus; (**C**) Bone calcium; (**D**) Bone phosphorus. Data are presented as mean ± SD (*n* = 12). ** indicates *p* < 0.01; *** indicates *p* < 0.001; **** indicates *p* < 0.0001.

Serum calcium concentration was significantly higher in the softened-bone group (90.235 ± 18.04 umol/mL) than in the hard-bone group (38.867 ± 14.36 umol/mL) (Figure 4A). In contrast, serum phosphorus concentration was significantly lower in the softened-bone group (1.474 ± 0.83 mmol/L) than in the hard-bone group (2.424 ± 0.54 mmol/L) (Figure 4B). In bone tissue, calcium concentration was lower in the softened-bone group (0.193 ± 0.02 mmol/g) than in the hard-bone group (0.266 ± 0.08 mmol/g) (Figure 4C). Similarly, bone phosphorus concentration was lower in the softened-bone group (0.532 ± 0.02 mmol/g) than in the hard-bone group (0.644 ± 0.08 mmol/g) (Figure 4D).

### 3.5. Comparative Histological Analysis of Bone Structures

**Figure 5:**
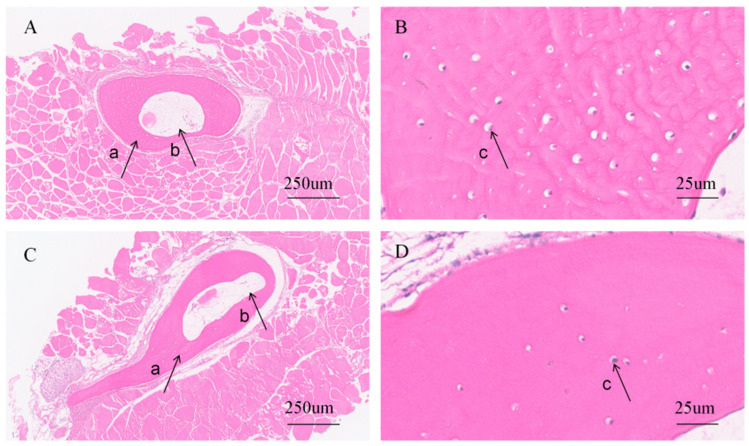
Comparison of rib morphology and structure between two groups. (**A**,**B**) H&E staining of ribs from hard-bone *C. c. rubrofuscus*; (**C**,**D**) H&E staining of ribs from softened-boned *C. c. rubrofuscus*; a. Cortical bone; b. Bone cavities; c. Osteocytes.

**Figure 6:**
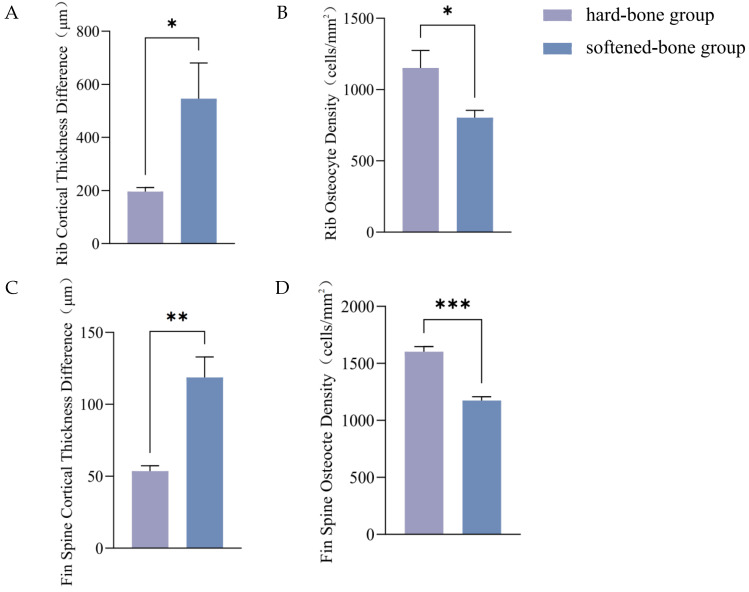
Comparison of cortical bone thickness differences and osteocyte densities. (**A**) Rib cortical thickness difference; (**B**) Rib osteocyte density; (**C**) Fin spine thickness difference; (**D**) Fin spine osteocyte density. Data are presented as mean ± SD (*n* = 3). * indicates *p* < 0.05; ** indicates *p* < 0.01. *** indicates *p* < 0.001.

Hematoxylin-eosin (H&E) staining of rib sections from both groups revealed typical bone structures, including cortical bone (a), bone cavities (b), and osteocytes (c) (Figure 5). The difference between the maximum and minimum cortical bone thicknesses was significantly greater in softened-bone *C. c. rubrofuscus* (509.75 ± 147.72 um) than in hard-bone individuals (196.03 ± 15.60 um), indicating more severe rib deformity in the softened-bone group (Figure 6A). In addition, osteocyte density in the ribs differed significantly between the two groups (*p* < 0.05), with values of 1152.23 ± 121.94 cells/mm^2^ in the hard-bone group and 802.75 ± 51.59 cells/mm^2^ in the softened-bone group (Figure 6B).

**Figure 7:**
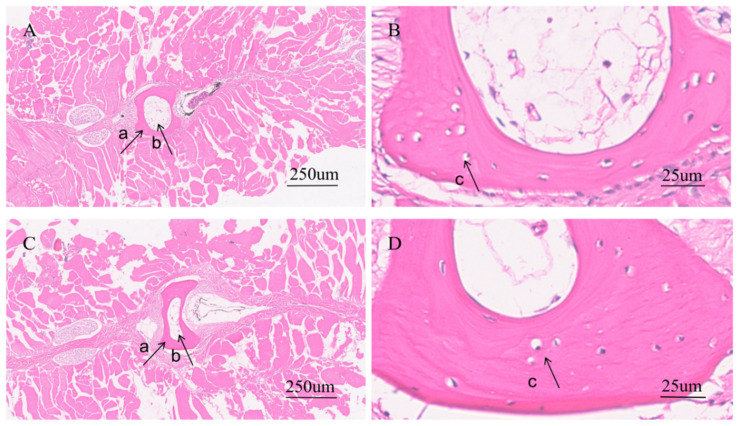
Comparative analysis of the tissue and structure of pterygiophore. (**A**,**B**) H&E staining of the radial fin bones of hard-bone *C. c. rubrofuscus*; (**C**,**D**) H&E staining of the radial fin rays of softened-bone *C. c. rubrofuscus*; a. Cortical bone; b. Bone cavities; c. Osteocytes.

Histological examination of the pterygiophores similarly revealed cortical bone, osteocytes, and bone cavities in both groups (Figure 7). The histological alterations observed in the pterygiophores of softened-bone *C. c. rubrofuscus* were consistent with those observed in the ribs. The difference between the maximum and minimum cortical bone thicknesses was significantly greater in the softened-bone group (109.47 ± 14.28 μm) than in the hard-bone group (53.50 ± 3.76 um) (Figure 6C, *p* < 0.05). In addition, osteocyte density was significantly lower in the softened-bone group (1174.29 ± 32.99 cells/mm^2^) than in the hard-bone group (1602.59 ± 44.63 cells/mm^2^) (Figure 6D, *p* < 0.05).

### 3.6. Comparative Analysis of Autophagy-Related Protein Levels and Relative Expression of Autophagy-Related Genes

**Figure 8:**
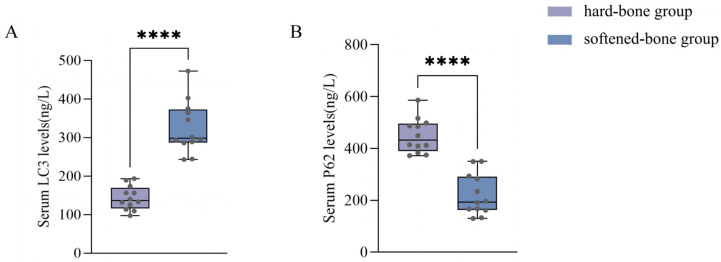
The serum level of Autophagy-Related Protein in two groups. (**A**) Serum LC3 levels; (**B**) Serum P62 levels. Data are presented as mean ± SD (*n* = 12). **** indicates *p* < 0.0001.

Considering that altered autophagy may contribute to reduced cell number, we further compared autophagy-related protein levels between the two groups. As shown in Figure 8, serum p62 concentrations were 449.13 ± 66.36 ng/L in the hard-bone group and 221.49 ± 79.30 ng/L in the softened-bone group, whereas serum LC3 concentrations were 143.42 ± 31.05 ng/L and 326.25 ± 68.03 ng/L, respectively. Compared with the hard-bone group, the softened-bone group showed extremely significant differences in both p62 and LC3 levels (*p* < 0.0001), characterized by a marked decrease in p62 and a marked increase in LC3 (Figure 8). These findings suggest that autophagy was markedly altered in the softened-bone group.

**Figure 9:**
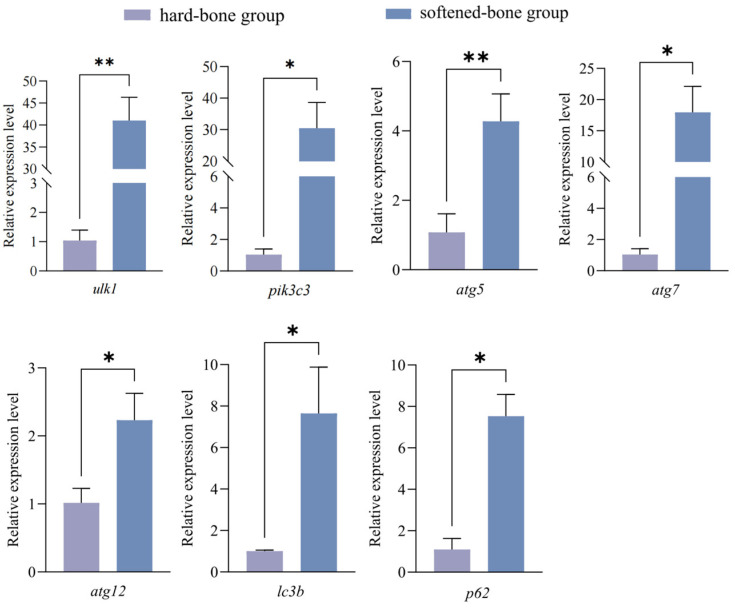
Relative expression levels of autophagy-related genes. Data are presented as mean ± SD (*n* = 12). * Indicates *p* < 0.05; ** indicates *p* < 0.01.

Meanwhile, qPCR analysis revealed that the relative expression levels of autophagy-related genes were significantly increased in the softened-bone group compared with the hard-bone group. As shown in Figure 9, the expression levels of *ulk1*, *pik3c3*, *atg5*, *atg7*, *atg12*, *lc3b*, and *p62* were upregulated in the softened-bone group. Together, these results indicate that the autophagy pathway was markedly dysregulated in the softened-bone group, suggesting that autophagy may play an important role in the regulation of bone formation and development in *C. c. rubrofuscus*.

## 4. Discussion

### 4.1. Effects of Bone Deformation on Growth

Bone deformities have been widely reported in farmed fish species, including *Barbus grypus*, *Trachinotus ovatus*, and *Scophthalmus maximus*. These abnormalities occur in various forms, such as mandibular curvature, shortened opercula, fin hyperplasia, anterior and posterior spinal protrusions, spinal curvature, and vertebral fusion [15,16,17]. Severe skeletal deformities can impair normal growth and may even lead to mortality [18]. For example, T. ovatus is prone to opercular deformity and shortening during early development, which expose the gill chamber, disrupt osmoregulation, increase disease susceptibility, and ultimately reduce stress tolerance and survival in aquaculture systems [19]. In cultured *Rachycentron canadum*, cranial deformities are mainly observed in the jaw and hyoid arches, which are key structures for food capture and ingestion. Individuals with such deformities show significantly reduced total length compared with hard-bone fish, indicating impaired feeding efficiency and growth suppression [20]. Likewise, spinal deformities in *Heterobranchus longifilis* and *Epinephelus fasciatus* significantly affect body weight and production yield [21,22]. In softened-bone *C. c. rubrofuscus*, however, deformities were mainly observed in the ribs and intermuscular spines, whereas no obvious abnormalities were detected in the cranial bones or vertebral column (Figure 3). Ribs mainly contribute to trunk support during locomotion and resistance to hydrodynamic stress [23]. Previous studies have shown that the absence of intermuscular bones in species such as *Ctenopharyngodon idella* and *Danio rerio* does not impair normal growth or development, suggesting that intermuscular spines play a limited role in growth regulation [24,25]. Consistent with these findings, morphometric measurements showed no significant differences between softened-bone and hard-bone *C. c. rubrofuscus* (Table 2 and Table 3). Together, these results suggest that deformities of the ribs and intermuscular spines have limited effects on feeding performance and somatic growth, which may explain the lack of significant morphological variation between the two groups.

### 4.2. Reduced Calcium and Phosphorus Levels Affect Bone Mineralization

Minerals are essential for fish growth, skeletal development, and metabolic regulation [26]. In this study, Alizarin Red S staining was visibly lighter in the softened-bone group, preliminarily suggesting a lower degree of mineralization than in the hard-bone group. To further evaluate mineralization status, in vivo X-ray imaging was used to quantify vertebral BMD. The softened-bone group showed significantly lower vertebral BMD (Figure 1 and Figure 2), confirming reduced mineral deposition. Calcium and phosphorus are the major mineral elements in fish and the principal inorganic components of the bone matrix. Approximately 99% of body calcium and 80% of body phosphorus are typically deposited as crystalline minerals in bones and scales, where they support skeletal development [27,28]. In the present study, softened-bone *C. c. rubrofuscus* exhibited significantly increased serum calcium and markedly decreased serum phosphorus, accompanied by substantially reduced calcium and phosphorus concentrations in bone tissue (Figure 4). Together, these findings indicate impaired mineral deposition and disrupted mineral homeostasis. In addition to dietary intake, environmental factors such as water chemistry can strongly influence mineral balance in fish [29,30,31,32]. For example, Acipenser fulvescens reared in different natural waters exhibits significant variation in calcium and magnesium levels [33]. Bone serves not only as a structural framework but also as a dynamic mineral reservoir. Under parathyroid hormone (PTH) signaling, bone resorption releases calcium into the circulation, whereas elevated serum calcium can promote bone deposition through calcitonin-associated pathways [34,35]. Therefore, the combination of reduced bone calcium and elevated serum calcium observed in softened-bone individuals suggests dysregulated calcium turnover and abnormal bone metabolism (Figure 4A). Previous studies have reported that wild yellow croaker has significantly higher vertebral BMD than farmed populations, whereas reduced BMD in farmed fish does not necessarily impair somatic growth [36]. Consistent with this pattern, the marked reductions in bone calcium and phosphorus in softened-bone *C. c. rubrofuscus* likely contributed directly to the decreased BMD and subsequent rib curvature and skeletal deformity. However, no significant differences in overall body morphology were detected between the softened-bone and hard-bone groups, suggesting that a certain degree of BMD reduction may be tolerated without obvious growth retardation in *C. c. rubrofuscus*.

### 4.3. Dysregulated Autophagy Is Associated with Osteocyte Reduction

Osteocytes are central regulators of skeletal development and remodeling and are interconnected through their dendritic processes [37]. By coordinating the activities of osteoblasts and osteoclasts, osteocytes participate in bone repair, regulation of bone mass, optimization of bone matrix composition, and adaptation to mechanical loading [38,39]. In the present study, H&E staining revealed marked deformities in the ribs and pterygiophores of softened-bone *C. c. rubrofuscus*, accompanied by a significant reduction in osteocyte density (Figure 5, Figure 6 and Figure 7). Previous studies have shown that a reduction in osteogenic cell populations can disturb mineral metabolism, impair bone mineralization, and ultimately reduce bone mass, BMD, and bone strength [38]. Studies in zebrafish have further demonstrated that impaired proliferation and differentiation of bone-associated cells can disrupt bone metabolic homeostasis, resulting in significant reductions in BMD and bone mass [39]. Similarly, studies in mouse models have shown that reduced osteocyte activity or osteocyte loss can induce osteoporosis-like phenotypes [40]. The maintenance of osteocyte number and function depends on multiple mechanisms involved in cellular homeostasis, among which autophagy is considered a key regulatory pathway.

Autophagy is an important mechanism for maintaining bone homeostasis and is closely associated with osteocyte survival in bone tissue [41]. Moderate activation of autophagy facilitates the clearance of damaged mitochondria and misfolded proteins, thereby supporting cell survival, whereas excessive activation may lead to over-degradation of intracellular components and ultimately trigger cell death [42,43,44,45]. Previous studies have shown that dysregulated autophagy can promote osteocyte apoptosis through mechanisms such as aggravated oxidative stress and mitochondrial dysfunction, thereby resulting in bone loss [45,46,47]. In the present study, compared with the hard-bone group, the softened-bone group of *C. c. rubrofuscus* exhibited significantly increased LC3 levels and significantly decreased p62 levels, suggesting dysregulated autophagy in the softened-bone group. Studies in pufferfish (*Tetraodontidae*) have shown that environmental stressors, such as toxicant exposure, can induce autophagy activation, as evidenced by increased LC3 levels and decreased p62 expression. This process is often accompanied by activation of the mitogen-activated protein kinase (MAPK) signaling pathway and an enhanced cellular stress response. Further studies in zebrafish have demonstrated that LC3 is a key marker of autophagosome formation, whereas p62 functions as a selective autophagy receptor that mediates cargo delivery and is subsequently degraded during the autophagic process. Excessive autophagy activation in zebrafish is likewise characterized by increased LC3 levels and decreased p62 expression, accompanied by enhanced apoptotic activity [48]. In the present study, the softened-bone group of *C. c. rubrofuscus* exhibited a similar pattern of LC3 upregulation and p62 downregulation, consistent with previous findings (Figure 8). These results suggest that autophagy was abnormally altered in the softened-bone group.

Meanwhile, the upregulated expression of multiple autophagy-related genes (*ulk1*, *pik3c3*, *atg5*, *atg7*, *atg12*, *lc3b*, and *p62*) in the softened-bone group suggests a robust activation of the autophagic program at the transcriptional level in bone cells. However, although decreased p62 protein levels together with increased LC3 abundance are generally regarded as indicators of enhanced autophagic activity, the concurrent elevation of *p62* mRNA does not conform to a simple linear model of autophagy activation. Rather than being contradictory, this discrepancy likely reflects the dynamic and multi-layered regulation of p62 [49,50,51,52]. Previous studies have shown that stress-responsive pathways, including ATF4/CHOP and NRF2 signaling, can induce the transcription of p62 and multiple ATG genes under nutrient deprivation, oxidative stress, or endoplasmic reticulum stress [53]. Therefore, the observed pattern—elevated *p62* mRNA coupled with reduced protein abundance—represents a state of enhanced autophagic turnover accompanied by compensatory transcriptional activation, rather than a true inconsistency. This suggests the presence of an autophagic imbalance characterized by activated initiation, increased substrate consumption, and stress-driven gene upregulation [49]. In this study, such a state is likely triggered by calcium–phosphorus imbalance. While this response may initially serve as a protective mechanism to maintain cellular homeostasis, its persistence may lead to autophagy dysregulation, ultimately impairing osteocyte function and bone mineralization [54]. Studies in mouse models have shown that osteoblast- or osteocyte-specific deletion of atg7 impairs autophagic function and leads to reduced bone mass, disruption of the osteocyte network, and compromised mechanical properties of bone, indicating that autophagy plays an important protective role in maintaining osteoblast survival and bone integrity [43]. In zebrafish, autophagy-related genes, including *atg5*, *atg7*, and *lc3*, are also highly expressed during skeletal development and osteogenic differentiation. Inhibition of these genes has been shown to impair osteoblast differentiation and bone matrix formation, suggesting that autophagy plays a conserved and essential regulatory role in skeletal development in teleosts [44].

Notably, disturbed calcium-phosphorus homeostasis and autophagic dysfunction were observed concurrently in the softened-bone group of *C. c. rubrofuscus*, suggesting a close association between mineral metabolism and autophagic regulation. Calcium and phosphorus are essential not only for bone mineralization but also for the regulation of osteocyte metabolism and signaling pathways [55]. As a major second messenger, Ca^2+^ r modulates autophagy initiation and lysosomal biogenesis through the CaMKKβ/AMPK/mTOR and calcineurin/TFEB pathways [56]. Under physiological conditions, appropriate Ca^2+^ signaling helps maintain autophagic homeostasis, whereas persistent calcium overload or disruption of calcium stores may result in abnormal autophagy activation or impaired autophagic flux [55,56]. On the other hand, phosphorus imbalance primarily affects autophagy through metabolic stress, leading to ATP deficiency, mitochondrial dysfunction, and reactive oxygen species (ROS) accumulation, which in turn activate AMPK-related pathways [57]. Persistent imbalance may further impair lysosomal function and autophagic flux, thereby shifting autophagy from a protective response to pathological dysregulation [58]. Importantly, calcium–phosphorus imbalance may interact with autophagic pathways through endoplasmic reticulum stress and apoptotic signaling, thereby affecting bone cell survival, differentiation, and mineralization [59]. Collectively, our findings suggested that disturbed calcium-phosphorus homeostasis in the softened-bone group may contribute not only to impaired mineral metabolism but also to autophagy-mediated dysregulation of bone cell function and impaired skeletal development.

## 5. Conclusions

Taken together, the present study demonstrates that the main manifestations of skeletal softening in *C. c. rubrofuscus* are rib curvature and reduced BMD, both of which are closely associated with disturbed calcium-phosphorus homeostasis and dysregulation of the autophagy pathway. The softened-bone group exhibited elevated serum Ca levels and decreased serum P levels, accompanied by increased LC3 protein levels and decreased p62 protein levels, suggesting abnormal alteration of the autophagic process. At the molecular level, the expression of autophagy-related genes, including *ulk1*, *pik3c3*, *atg5*, *atg7*, *atg12*, *lc3b*, and *p62*, was significantly upregulated in bone tissue, further supporting the occurrence of autophagic dysregulation. Notably, the concomitant upregulation of *p62* mRNA and downregulation of p62 protein suggests that the softened-bone group may exhibit a more complex disturbance in autophagic regulation, rather than a simple increase in autophagic flux. Mechanistically, disturbed calcium-phosphorus metabolism together with abnormal autophagy in the softened-bone group may contribute to osteocyte loss, ultimately leading to reduced BMD and impaired skeletal development. Notably, skeletal softening and deformity, as well as reduced BMD, did not significantly alter external body morphology, providing a practical basis for the selective breeding of high-quality cyprinid fish with a softened-bone phenotype. The pronounced inter-individual variation in bone traits further suggests that calcium-phosphorus absorption efficiency and autophagy-regulatory capacity may have underlying genetic determinants.

## Figures and Tables

**Table 1:** Formulation and proximate composition of experimental diets (%).

Ingredient	Nutritional Information
Fish meal	10.00
Soybean meal	15.00
Peanut meal	15.00
Cottonseed meal	12.00
Rapeseed meal	10.00
Wheat flour	20.00
Rice bran	7.86
Soybean oil	2.30
vitamin complex	1.00
Composite mineral	1.50
Total	100.00
Proximate composition	
Crude protein	31.42
Crude lipid	5.80
Crude ash	4.30
Ca	1.34
p	0.62
Ca/P	2.16

**Table 2:** Primers used in the study.

Gene Name	Primer Name	Sequence (5′-3′)	Tm	Product Length
*ulk1*	ulk_f	CAGGACCTCCGATTGTTTTATG	60	82
	ulk_r	GGTGTCTGAGATGGCTGGAAGT		
*pik3c3*	pik_f	GTGGAGGGTGATGGCAGTG	60	117
	pik_r	CATGTGACCCTGACGATGAGC		
*atg5*	atg5_f	CTCCTCCCACGGGTCAGTT	60	147
	atg5_r	CAAAACGCCAATGGGATAGTG		
*atg7*	atg7_f	CCACAGCAGCAGCCACCAT	60	95
	atg7_r	GGCGACCACATCGTTACAGAAG		
*atg12*	atg12_f	AATCAGTCATTTGCTCCATC	60	97
	atg12_r	GCCAGATGGGGCAGAGTAG		
*lc3b*	lc3_f	GACCACGTAAACATGAGCGAAC	60	88
	lc3_r	CGTTGACGAGCAGGAAGAAAG		
*p62*	p62_f	TACAGCATTTCATCCTGCCTCC	60	143
	p62_r	CCTCGTCACTGCCTTGTTCTTT		
*GAPDH*	GAPDH_f	AGCTCAATGGCAAGCTTACTGG	60	184
	GAPDH_r	GTGGATACCACCTGGTCCTCTG		

**Table 3:** The mean value of the morphological traits between two groups of *C. c. rubrofuscus*.

Traits	Group	
	Hard-Bone Group	Softened-Bone Group
Weight (g)	100.18 ± 13.14	97.10 ± 10.54
*T*_L_ (cm)	16.09 ± 1.17	16.26 ± 1.13
*S*_L_ (cm)	6.73 ± 0.77	6.21 ± 0.64
*B*_D_ (cm)	6.27 ± 0.64	6.10 ± 0.51
*B*_W_ (cm)	3.53 ± 0.32	3.25 ± 0.35
*H*_L_ (cm)	4.03 ± 0.23	3.98 ± 0.26
*H*_H_ (cm)	3.68 ± 0.31	3.48 ± 0.28
*CP*_D_ (cm)	2.64 ± 0.46	2.44 ± 0.31
*CP*_L_ (cm)	2.01 ± 0.52	1.80 ± 0.16
*CF*_L_ (cm)	3.33 ± 0.74	4.25 ± 0.77

**Table 4:** The eight morphometric characteristics between two groups of *C. c. rubrofuscus*.

Morphometric Ratios	Group	
	Hard-Bone Group	Softened-Bone Group
*T*_L_/*S*_L_	2.40 ± 0.17	2.63 ± 0.20
*B*_W_/*S*_L_	0.53 ± 0.04	0.54 ± 0.04
*B*_D_/*S*_L_	0.93 ± 0.05	0.98 ± 0.07
*H*_L_/*S*_L_	0.61 ± 0.07	0.64 ± 0.04
*H*_H_/*B*_D_	0.91 ± 0.08	0.88 ± 0.09
*CP_L_*_/_ *S*_L_	0.30 ± 0.06	0.29 ± 0.02
*CP_D_*/*CP_L_*	1.37 ± 0.31	1.36 ± 0.21
*CF*_L_/*S*_L_	0.50 ± 0.14	0.69 ± 0.15

## Data Availability

The data presented in this study are available upon request from the corresponding author.

## References

[1] Du A.F., Li W.H., Wei L.J., Mo F.L., Lu Y.D., Liu K., Ye X.C., Zhang Y.L., Zhang S. (2024). Nutritional component analysis and quality evaluation of *Procypris merus* in Guangxi. Anhui Nongye Kexue.

[2] Zhu H.P., Ma D.M., Li J.Z., Fu Y., Han L.Q. (2021). Common carp (*Cyprinus carpio*) ‘Ruyuan No. 1’. Zhongguo Shuichan.

[3] Cahu C., Zambonino Infante J., Takeuchi T. (2003). Nutritional components affecting skeletal development in fish larvae. Aquaculture.

[4] Zhang S.J. (2008). Effects of various nutrients on bone health in fish. Siliao Yanjiu.

[5] Lyu X.J. (2018). Growth Characteristics, Osteological Ontogeny and Deformity in Larval and Juvenile *Scophthalmus maximus* and *Epinephelus lanceolatus*. Ph.D. Thesis.

[6] Ma H., Zhuang Z.M., Liu S.F., Ma J., Wang X.L. (2011). Skeletal deformities in the larvae and juveniles of cultured tongue sole (*Cynoglossus semilaevis*). Zhongguo Shuichan Kexue.

[7] Lewis M.L., Lall P.S., Witten E.P. (2004). Morphological descriptions of the early stages of spine and vertebral development in hatchery-reared larval and juvenile Atlantic halibut (*Hippoglossus hippoglossus*). Aquaculture.

[8] Zheng P.L., Ma Z.H., Guo H.Y., Zhang N., Zhang D.C., Jiang S.G. Osteological ontogeny and malformations in larval and juvenile of rearing golden pompano (*Trachinotus ovatus*). Proceedings of the Abstract Book of the Annual Conference of the Chinese Fisheries Society.

[9] Cheng D.C. (2016). Ontogenetic Development of Crimson Snapper (*Lutjanus erythropterus*). Master’s Thesis.

[10] Han M.Y., Zhou S.J., Yang R., Hu J., Ma Z.H. (2021). Histopathology and molecular characterization of the skeletal tiaaues of golden pompano (*Trachinotus ovatus*) larvae under temperature stress. Nanfang Nongye Xuebao.

[11] Li X.Q., Wang Z.Y., Ma J.H., Zhao J.J. (1993). Preliminary study on osteomalacia of intensively pond-cultured common carp (*Cyprinus carpio*). Zhongguo Shouyi Kexue.

[12] Huang J., Huang K., Jie B.F., Jiang L.Y., Mo C.Q., Yu K., Guo R.J., Wu Y.T. (2024). Effects of dietary calcium and phosphorus levels on growth performance, serum biochemical indices, skeleton and liver histomorphology of *Cyprinus carpio var*. Feed Ind..

[13] Sun X., Zhang R., Chen H., Du X., Chen S., Huang J., Liu M., Xu M., Luo F., Jin M. (2020). Fgfr3 mutation disrupts chondrogenesis and bone ossification in zebrafish model mimicking CATSHL syndrome partially via enhanced Wnt/β-catenin signaling. Theranostics.

[14] Fjelldal P.G., Hansen T. (2010). Vertebral deformities in triploid Atlantic salmon (*Salmo salar* L.) underyearling smolts. Aquaculture.

[15] Malekpouri P., Mesbah M., Rezaie A. (2015). Aetiology of skeletal deformity in a Barbus grypus (Heckel, 1843) fish: Clinical and radiological studies. Comp. Clin. Pathol..

[16] Ma Z.H., Guo H.Y., Zhang D.C., Fu M.J., Zhang N., Jiang S.G. (2016). Osteological ontogeny and malformations in larval and juvenile golden pompano *Trachinotus ovatus* (Linnaeus, 1758). Aquac. Res..

[17] Lv X., Xu S., Liu Q., Wang X., Yang J., Song Z., Li J. (2018). Osteological ontogeny and allometric growth in larval and juvenile turbot (*Scophthalmus maximus*). Aquaculture.

[18] Huang C.T., Xiao Y., Luo Z.Z. (2013). Research progress on skeletal development in larval and juvenile fish. Dangdai Shuichan.

[19] Wu Y.W., Ma Q., Huang D.H., Wang L.Y., Zhou Q.L., Ran C.L., Chen G. (2025). Effect of opercular deformity on morphological and transcriptomic profiling of gills in golden pompano (*Trachinotus ovatus*). Aquac. Rep..

[20] Mao F.F., Chen G., Ma Q., Zhou Q.L., Shi G., Huang J.S., Kuang J.H. (2022). Skeletal deformities in the juveniles of cultured cobia (*Rachycentron canadum*). Yuye Kexue Jinzhan.

[21] Suleiman B., Maruff L., Oniye S.J. (2015). Radiographic studies on morphological anomalies in artificially spawned *Heterobranchus longifilis* (Valenciennes, 1840) F1 generation. Sokoto J. Vet. Sci..

[22] Kong X.D., Liu L., Li Y.L., Yu H.H., Zhai J.M., Pang Z.F., Xu W.T., Chen C. (2016). Nutritional values of 2-year-old cultured *Epinephelus akaara* and causes of its common deformities. Prog. Fish Sci..

[23] Jiao Y.Y., Okada M., Hara E.S., Xie S.C., Nagaoka N., Nakano T., Matsumoto T. (2020). Micro-architectural investigation of teleost fish rib inducing pliant mechanical property. Materials.

[24] Nie C.H., Wan S.M., Tomljanovic T., Treer T., Hsiao C.D., Wang W.M., Gao Z.X. (2021). Comparative analysis of embryonic muscle development in wildtype Zebrafish and its intermuscular bone deficiency mutant. Gene.

[25] Qian Y.Q., Zheng J.B., Xu X.F., Luo C. (2015). Normally grown and developed intermuscular bone-deficient mutant in grass carp, *Ctenopharyngodon idellus*. Chin. Sci. Bull..

[26] Huang F. (2011). Shuisheng Dongwu Yingyang Yu Siliao Xue [Aquatic Animal Nutrition and Feed Science].

[27] Lin H.R. (1999). Yu Lei Sheng Li Xue [Fish Physiology].

[28] Yao Y.F. (2012). Dietary available phosphorus and Ca/P requirements for juvenile and adult GIFT strain of Nile tilapia (*Oreochromis niloticus*). Master’s Thesis.

[29] Song J.Y., Wang L., Zhang C.X., Song K., Hu S.C., Zhang L. (2015). Effects of dietary calcium levels on regulation of serum osmolality and ion levels in Japanese seabass (*Lateolabrax japonicus*) in freshwater. Dalian Haiyang Daxue Xuebao.

[30] Zhao C.Y., Zhou H.Q., Chen J.M., Xu P., Li H.X. (2008). Effects of dietary levels of phosphorus on growth and biochemical composition of *Hemibarbus maculates* Bleeker. Shanghai Haiyang Daxue Xuebao.

[31] Hu P.L. (2019). Effects of Dietary Calcium and Phosphorus Levels on Growth, Calcium and Phosphorus Digestion, Absorption and Deposition of Spotted Sea Bass (*Lateolabrax maculatus*). Master’s Thesis.

[32] Martens L.G., Fjelldal P.G., Lock E.J., Witten P.E., Hansen T.J., Waagbø R. (2022). Mineral balance and bone formation in fast-growing Atlantic salmon parr (*Salmo salar*) in response to dissolved metabolic carbon dioxide and restricted dietary phosphorus supply. Aquaculture.

[33] Genz J., Hicks R.N., Prabhu A., Schrama J.W. (2021). Response in growth, scute development, and whole-body ion composition of *Acipenser fulvescens* reared in water of differing chemistries. Animals.

[34] Tao B., Wang O., Xing X.P., Xia W.B. (2025). Certain key questions in the diagnosis and treatment of primary osteoporosis and other disorders related to abnormal calcium and phosphorus metabolism. Chin. Med. J..

[35] Song Y.T., Wu Y. (2020). Calcitonin synthesis, secretion and physiological effects. Zhongguo Guzhi Shusong Zazhi.

[36] Wang Y., Zhao J.L., Ke Q.Z., Liu J.F., Chen J., Weng H.S., Han K.H. (2016). Comparison of bone microstructures in cultured and wild stocks of large yellow croaker (*Larimichthys crocea*) using micro CT scanning. J. Ocean Univ. China.

[37] Schaffler M.B., Cheung W.Y., Majeska R., Kennedy O. (2014). Osteocytes: Master orchestrators of bone. Calcif. Tissue Int..

[38] Baschant U., Altamura S., Steele-Perkins P., Muckenthaler M.U. (2020). Disruption of the hepcidin/ferroportin regulatory circuitry causes low axial bone mass in mice. Bone.

[39] Bonewald L.F. (2011). The amazing osteocyte. J. Bone Miner. Res..

[40] Yan Z.D. (2025). Study on the Mechanism of Bone Stress Signal Transduction Based on Osteocyte NFATc3. Doctoral Dissertation.

[41] Klein-Nulend J., Bakker A.D., Bacabac R.G., Vatsa A., Weinbaum S. (2013). Mechanosensation and transduction in osteocytes. Bone.

[42] Klionsky D.J., Abdel-Aziz A.K., Abdelfatah S., Abdellatif M., Abdoli A., Abel S., Abeliovich H., Abildgaard M.H., Abudu Y.P., Acevedo-Arozena A. (2016). Guidelines for the use and interpretation of assays for monitoring autophagy (3rd edition). Autophagy.

[43] Mizushima N., Yoshimori T. (2007). How to interpret LC3 immunoblotting. Autophagy.

[44] Wang Y., Liu N., Lu B., Zhang Q., Zhang Y., Li J., Li Y., Liu X., Chen Y., Zhao Y. (2022). Autophagy in bone remodeling: A regulator of oxidative stress. Front. Endocrinol..

[45] Onal M., Piemontese M., Xiong J., Han L., Ye S., Komatsu M., Selig M., Weinstein R.S., Jilka R.L., O’Brien C.A. (2013). Suppression of autophagy in osteocytes mimics skeletal aging. J. Biol. Chem..

[46] Moss J.J., Hammond C.L., Lane J.D. (2020). Zebrafish as a model to study autophagy and its role in skeletal development and disease. Histochem. Cell Biol..

[47] Ru J., Wang Y. (2020). Osteocyte apoptosis: The roles and key molecular mechanisms in resorption-related bone diseases. Cell Death Dis..

[48] Tang Z.X., Wang H.Z., Peng C., Yin S.W., Wang T. (2026). Molecular characterization of LC3, Beclin-1 and P62 and their response patterns under low temperature and copper ion exposure in *Takifugu fasciatus*. Anim. Adv..

[49] B’Chir W., Maurin A.C., Carraro V., Averous J., Jousse C., Muranishi Y., Parry L., Stepien G., Fafournoux P., Bruhat A. (2013). The eIF2α/ATF4 pathway is essential for stress-induced autophagy gene expression. Nucleic Acids Res..

[50] Sahani M.H., Itakura E., Mizushima N. (2014). Expression of the autophagy substrate SQSTM1/p62 is restored during prolonged starvation depending on transcriptional upregulation and autophagy-derived amino acids. Autophagy.

[51] Klionsky D.J., Petroni G., Amaravadi R.K., Baehrecke E.H., Ballabio A., Boya P., Bravo-San Pedro J.M., Cadwell K., Cecconi F., Choi A.M.K. (2021). Guidelines for the use and interpretation of assays for monitoring autophagy (4th edition). Autophagy.

[52] Mathai B.J., Meijer A.H., Simonsen A. (2017). Studying autophagy in zebrafish. Cells.

[53] Jain A., Lamark T., Sjøttem E., Larsen K.B., Awuh J.A., Øvervatn A., McMahon M., Hayes J.D., Johansen T. (2010). p62/SQSTM1 is a target gene for transcription factor NRF2 and creates a positive feedback loop by inducing antioxidant response element-driven gene transcription. J. Biol. Chem..

[54] Nollet M., Santucci-Darmanin S., Breuil V., Al-Sahlanee R., Cros C., Topi M., Momier D., Samson M., Pagnotta S., Cailleteau L. (2014). Autophagy in Osteoblasts Is Involved in Mineralization and Bone Homeostasis. Autophagy.

[55] Høyer-Hansen M., Bastholm L., Szyniarowski P., Campanella M., Szabadkai G., Farkas T., Bianchi K., Fehrenbacher N., Elling F., Rizzuto R. (2007). Control of macroautophagy by calcium, calmodulin-dependent kinase kinase-beta, and Bcl-2. Mol. Cell.

[56] Tong Y., Song F. (2015). Intracellular calcium signaling regulates autophagy via calcineurin-mediated TFEB dephosphorylation. Autophagy.

[57] Fujimura R., Yamamoto T., Takabatake Y., Isaka Y., Yoshida S., Takahashi A., Namba-Hamano T., Minami S., Takahashi Y., Matsui I. (2020). Autophagy protects kidney from phosphate-induced mitochondrial injury. Biochem. Biophys. Res. Commun..

[58] Nguyen N.T., Nguyen T.T., Park K.-S. (2022). Oxidative Stress Related to Plasmalemmal and Mitochondrial Phosphate Transporters in Vascular Calcification. Antioxidants.

[59] Liu F., Fang F., Yuan H., Yang D., Chen Y., Williams L., Goldstein S.A., Krebsbach P.H., Guan J.-L. (2013). Suppression of Autophagy by FIP200 Deletion Leads to Osteopenia in Mice through the Inhibition of Osteoblast Terminal Differentiation. J. Bone Miner. Res..

